# Cleaning Efficacy of Air Polishing on Tobacco‐Stained Resin Composite: An In‐Vitro Study

**DOI:** 10.1002/cre2.70406

**Published:** 2026-07-08

**Authors:** Lukas Sigwart, Vera Wiesmüller, Ines Kapferer‐Seebacher

**Affiliations:** ^1^ Department of Dental and Oral Medicine and Cranio‐Maxillofacial and Oral Surgery University Hospital for Conservative Dentistry and Periodontology, Medical University of Innsbruck Innsbruck Austria

**Keywords:** professional tooth cleaning, profilometry, resin composite, smoking, spectrophotometry, tooth discolorations

## Abstract

**Objective:**

To evaluate the efficacy of air‐polishing on color change and surface roughness of resin composite and enamel specimens after standardized cigarette smoke exposure.

**Methods:**

Sixty composite/enamel specimens were exposed daily to cigarette smoke in an automated chamber over four 14‐day cycles (five cigarettes/day). After each cycle, specimens were cleaned using air‐polishing with erythritol or sodium bicarbonate powder, or a rubber cup and pumice (control). Color changes (Δ*E*) were assessed spectrophotometrically, and surface roughness was measured profilometrically before and after each cycle.

**Results:**

All cleaning methods significantly reduced tobacco staining on composite and enamel; however, none restored the original color. After four cycles, median [IQR] Δ*E* values for resin composites did not differ significantly among groups (erythritol: 26.17 [21.62–27.52]; sodium bicarbonate: 10.28 [7.44–27.77]; control: 24.56 [19.22–26.77]; *p* > 0.05). Surface roughness increased significantly in all groups, with the greatest increase following sodium bicarbonate air‐polishing (*p* < 0.05). Luting gap roughness increased significantly with sodium bicarbonate in comparison with erythritol and the control group (*p* < 0.001). Repeated air‐polishing did not promote further tobacco stain accumulation.

**Conclusions:**

Erythritol air‐polishing effectively reduced discoloration with minimal surface alteration, whereas sodium bicarbonate caused significant roughening.

**Clinical Significance:**

Cigarette smoke causes clinically perceptible composite discoloration that cannot be completely eliminated. Erythritol represents a safer polishing option than sodium bicarbonate.

**Abbreviations**
AT: acceptability threshold
MAP: minimal abrasive powder
MAPD: minimal abrasive powder device
PT: perceptibility threshold
Ra: average arithmetic roughness
Rpm: rotations per minute
*R*_t_
: total roughness
*R*_z_
: average roughness

## 1 Introduction

The utilization of resin‐based composites for the direct restoration of anterior and posterior teeth is well established in restorative dentistry, owing to their favorable esthetic, physical, and mechanical properties. In the domain of clinical restorative dentistry, proper finishing and polishing are of paramount importance, as they enhance the esthetics and durability of restorations (Roeder and Powers 2004). The surface roughness of resin composites is affected by the type of composite material (e.g., nanohybrid, microhybrid) and its porosity, along with the polishing instruments and techniques used (Sarac et al. 2006; Yap et al. 1997). An increase of surface roughness, whether on dental hard tissues or on direct restorations, plays a crucial role in the risk of gingival inflammation and secondary caries by promoting increased plaque accumulation (Li et al. 1985; Bollenl et al. 1997; Jefferies 1998). Demarco et al. (2023) reported in their review that the most common reasons for the failure of resin composite restorations are esthetic concerns in anterior teeth and secondary caries in posterior teeth.

Exposure to cigarette smoke can result in both extrinsic and intrinsic discolorations of oral hard tissues. Extrinsic staining occurs on dental surfaces, primarily due to the deposition of tar, nicotine, and other pigmented chemicals. In contrast, intrinsic discoloration arises when nicotine and other reactive compounds penetrate the enamel and dentine, leading to yellowing from within the tooth structure itself. Both types of discoloration can also affect resin composite restorations, which may absorb pigments and become stained over time (Karanjkar et al. 2023; de Geus et al. 2018). The issue of tobacco staining is not merely a matter of esthetics. It is widely acknowledged that it is also a significant risk factor for the accumulation and alterability of dental plaque (Conte et al. 2023; Zini et al. 2011; Robbins and Ali 2022; Macgregor et al. 1985; Socransky et al. 1998). Consequently, comprehensive prophylactic care comprising professional tooth cleaning to remove stains, extrinsic discolorations, and dental biofilm, oral hygiene instructions, and the application of fluoride and/or other remineralizing agents are essential for ensuring the long‐term success of direct restorations.

Air polishing offers simplified handling and requires less manual dexterity compared to conventional polishing methods. Moreover, when appropriate powders are used, air polishing is considered gentle on both hard and soft dental tissues (Petersilka, Bell et al. 2003; Petersilka, Steinmann et al. 2003; Atkinson et al. 1984; Berkstein et al. 1987; Wolgin et al. 2021). The most commonly utilized powders in this context are erythritol, sodium bicarbonate, and glycine, all of which have been shown to exhibit equivalent cleaning efficacy (Güler et al. 2010, 2011). Recent clinical studies have demonstrated that air polishing with erythritol powder provides superior capacity for biofilm removal compared to rubber cup polishing (Wolgin et al. 2021; Petersilka 2011; Fu et al. 2021). This approach has also been associated with significantly reduced treatment times and improved short‐term outcomes in the resolution of gingivitis (Wolgin et al. 2021; Fu et al. 2021; Mensi et al. 2022). Furthermore, in long‐term periodontal maintenance, minimal abrasive powder devices (MAPDs) have been shown to be safe, yielding clinical outcomes comparable to conventional mechanical debridement (Petersilka et al. 2021).

In a recent study, the authors evaluated the efficacy of various polishing techniques on tobacco‐stained dental hard tissues and found that erythritol air polishing removed smoker's stains as effectively as air polishing with sodium bicarbonate or pumice paste polishing, without affecting the surface roughness of the teeth (Sigwart et al. 2024). Furthermore, tobacco stains did not reappear more quickly or intensely following air polishing compared to conventional rubber cup and paste polishing (Sigwart et al. 2024). We wonder whether the repeated use of air‐polishing can also restore the original color of tobacco‐stained resin composite restorations without altering the surface characteristics, and whether the regrowth of tobacco stains is affected by the selected procedure. Therefore, the aim of the present study was to compare the color changes and surface roughness of standardized specimens composed of composite and enamel, which were repeatedly exposed to cigarette smoke and cleaned in vitro, using either erythritol air‐polishing, sodium bicarbonate air‐polishing, or rubber cup and pumice stone paste.

The null hypothesis was that there would be no difference in color change on resin composite restorations between the conventional polishing with rubber cup and pumice stone, compared to air‐polishing with erythritol or sodium bicarbonate after repeated use.

## 2 Materials and Methods

### 2.1 Ethical Approval

The Ethics committee of the Medical University of Innsbruck, Austria, approved the study (Biobank of extracted teeth, EK1178/2022 for the storage and scientific use of extracted teeth and EK1027/2023 for the approval of our trials). Patients who donated their extracted teeth for scientific research signed a consent form before tooth preservation. The study was conducted per the Helsinki Declaration of 1964 and its later amendments.

### 2.2 Samples

Thirty extracted permanent molars from non‐smokers were obtained from the Biobank of extracted teeth, Medical University of Innsbruck, Austria to create 60 samples with a baseline color range of A2 (VITA classical shade guide; VITA Zahnfabrik; Bad Säckingen, Germany). All teeth were sound and extracted due to medical reasons. Before preservation, teeth were cleaned by rinsing with 3%‐hydrogen peroxide for 15 min and stored in a 1% thymol solution (University pharmacy Innsbruck, Innsbruck, Austria). The crowns were first separated from the roots and then sectioned in an apico‐coronal direction into samples measuring 6 × 6 × 2 mm^3^—matching the diameter of the spectrophotometer *VITA Easyshade* V (VITA Zahnfabrik; Bad Säckingen, Germany)—using a diamond‐coated rotating separation disc (Komet, Lemgo, Germany) operated at 10,000 rpm. On the straight cut edge of the enamel samples, resin composite fillings of equal size were placed. According to the manufacturer's instructions, the enamel was etched with 38% phosphoric acid for 30 s, bonded with a one‐bottle adhesive system (Adhese universal, Ivoclar Vivadent, Schaan, Liechtenstein) for 30 s, and light cured for 20 s. The nanohybrid resin composite IPS Empress Direct A2 enamel (Ivoclar Vivadent, Schaan, Liechtenstein) was applied and light cured for 30 s. Finally, the restorations were sequentially polished with polishing discs (Sof‐Lex, 3M, Neuss, Germany), progressing from coarse (grain size: 60 µm) to fine grits (grain size: 3 µm), until the luting margins were clinically satisfactory and the restoration surfaces exhibited a smooth, uniform finish free of irregularities.

### 2.3 Color and Roughness Measurement

Color and surface roughness measurements followed the protocol described in detail in our previous study (Sigwart et al. 2024). Color measurements were conducted with the spectrophotometer *VITA Easyshade* V (VITA Zahnfabrik; Bad Säckingen, Germany), a cordless portable, battery‐operated, contact‐type spectrophotometer with a measuring range from 400 to 700 nm (Klotz et al. 2022; Kielbassa et al. 2009). The CIELAB formula was used to calculate the shade difference Δ*E* between two different measurements (Cheung and Rhodes 2023). In brief, the color is based on the L*a*b‐color space and calculated with the formula $E=\sqrt{L^{2}+a^{2}+b^{2}}$, and the color difference between two measurements by the formula ∆$E=\sqrt{∆L^{2}+{∆a}^{2}+∆b^{2}}$. The greater the deviation ∆*E*, the greater is the color difference. For a more comprehensive clinical understanding of our results, the color *E* was transmitted to the VITA classical shade guide with the same VITA Easyshade V, and the color difference was evaluated against the perceptibility threshold (PT) and the acceptability threshold (AT). The PT represents the smallest color difference that can be detected by the human eye under controlled conditions, whereas the AT defines the maximum color difference considered clinically acceptable by observers. As recommended by Rioseco and Wagner (2021), AT was set at Δ*E* = 2.7 and the PT at Δ*E* = 1.2 (Paravina et al. 2015; Khashayar et al. 2014).

Surface roughness was evaluated using a profilometer (Talysurf; Taylor Hobson AMETEK; Warrenville, IL, USA), where a tactile sensor fumbles a defined length of 2 mm (Cut off: 0.25 mm, measurement‐velocity: 0.50 mm/s) and creates a surface profile. The profiles' roughness parameters, total roughness *R*_t_ (difference between the highest peak and the lowest valley), and average roughness *R*_z_ were calculated separately for enamel and resin composite surfaces. *R*_t_ was additionally used to analyze the extent of the luting gaps. All samples were secured in a standardized mount to allow repeated measurements of the same surface under reproducible conditions.

### 2.4 Automated Smoking Chamber and Cleaning Procedure

**Figure 1:**
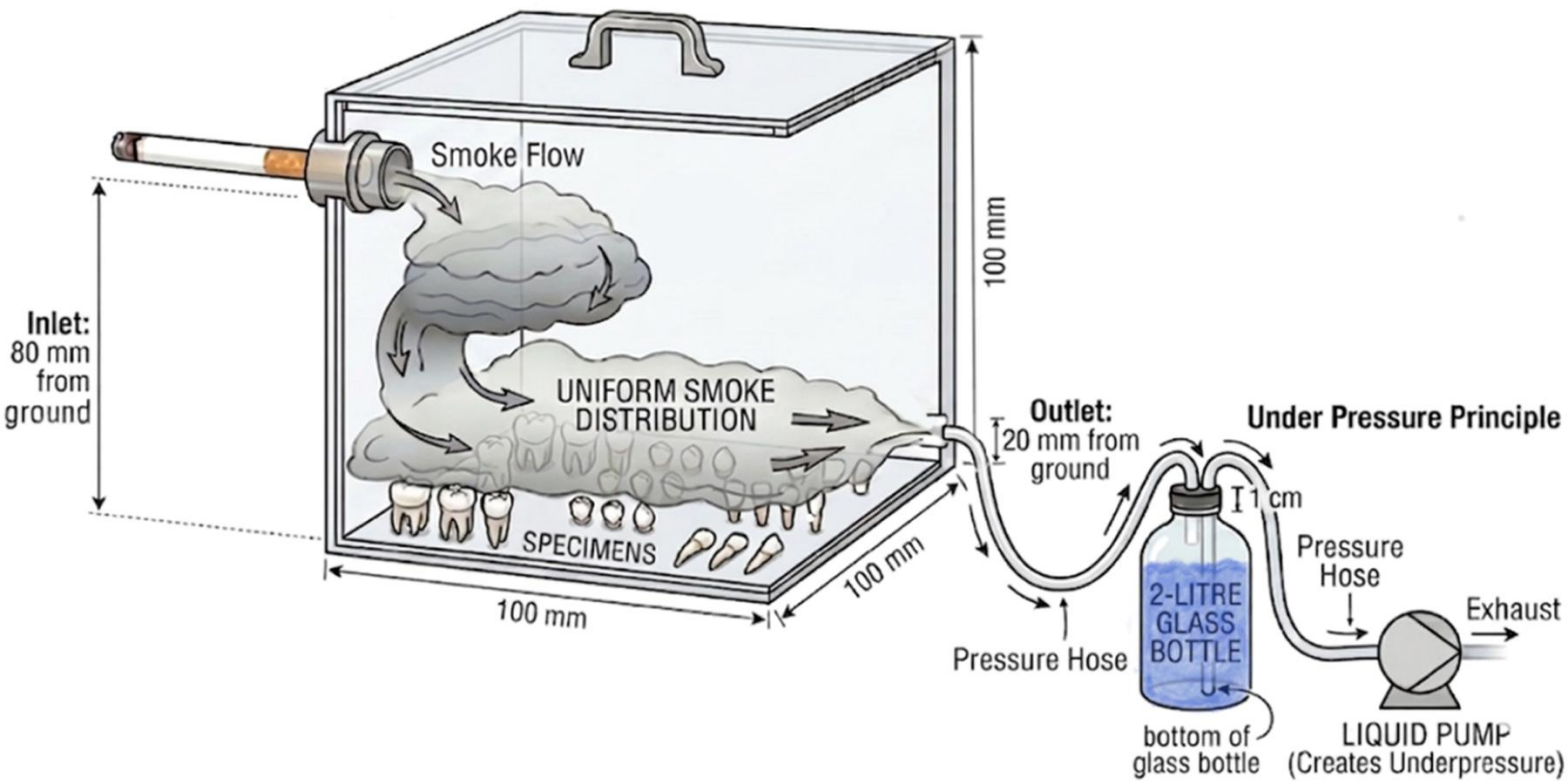
Graphical representation of the smoking chamber. The liquid pump creates an underpressure on the outlet of the smoking chamber that is used in order to combust the cigarette at the inlet. The inlet was designed at the top of the smoking chamber so that the cigarette smoke can flow down on the ground of the smoking chamber to expose all specimen homogenously, independent of the position within the chamber.

As described previously, an automated smoking chamber was employed to produce standardized tooth discolorations under controlled in vitro conditions simulating typical human smoking (see Figure 1) (Sigwart et al. 2024). In brief, the chamber design consists of a 3D‐printed cube (100 × 100 × 100 mm^3^) with hermetically sealed inlet and outlet hose connectors (Camozzi, Hall, Austria). Cigarettes were inserted into the inlet connector and combusted using a liquid pump (35 mL/s, 2 L water; UNILAB, Innsbruck, Austria) for 30 s, ensuring uniform smoke distribution within the chamber, allowing all samples to be exposed to the same smoke concentration. After each cigarette, the chamber was ventilated to remove residual smoke.

The experimental protocol involved four repetitions of a 14‐day smoking cycle with five cigarettes every day and professional tooth cleaning after each cycle. To mimic oral conditions and to establish a pellicle layer on the samples, all samples were stored in artificial saliva (Pickering laboratories; Mountain View, CA, USA) for 48 h prior to the first smoking cycle and for the entirety of the study period, with the exception of the exposure time in the smoking chamber and during the measurement and cleaning procedures. After each 14‐days smoking cycle, test group I was cleaned with air‐polishing using erythritol powder, test group II with air‐polishing using sodium bicarbonate, and the control group with rubber cup and pumice stone. In both air‐polishing groups, samples were cleaned 10 s with the airflow device Prophylaxis master and the Airflow MAX handpiece (both by E.M.S.; Nyon, Switzerland) in an angle of 45° with a working distance of 2 mm between handpiece outlet and surface with the settings of a waterflow of 60 mL/min with 2 g/min of the appropriate powder by a dynamic pressure of 3.1 bar of the airflow and in circumferential movements. All cleaning procedures were performed by two independent professionals which were calibrated before the trials by the head of the study.

**Figure 2:**
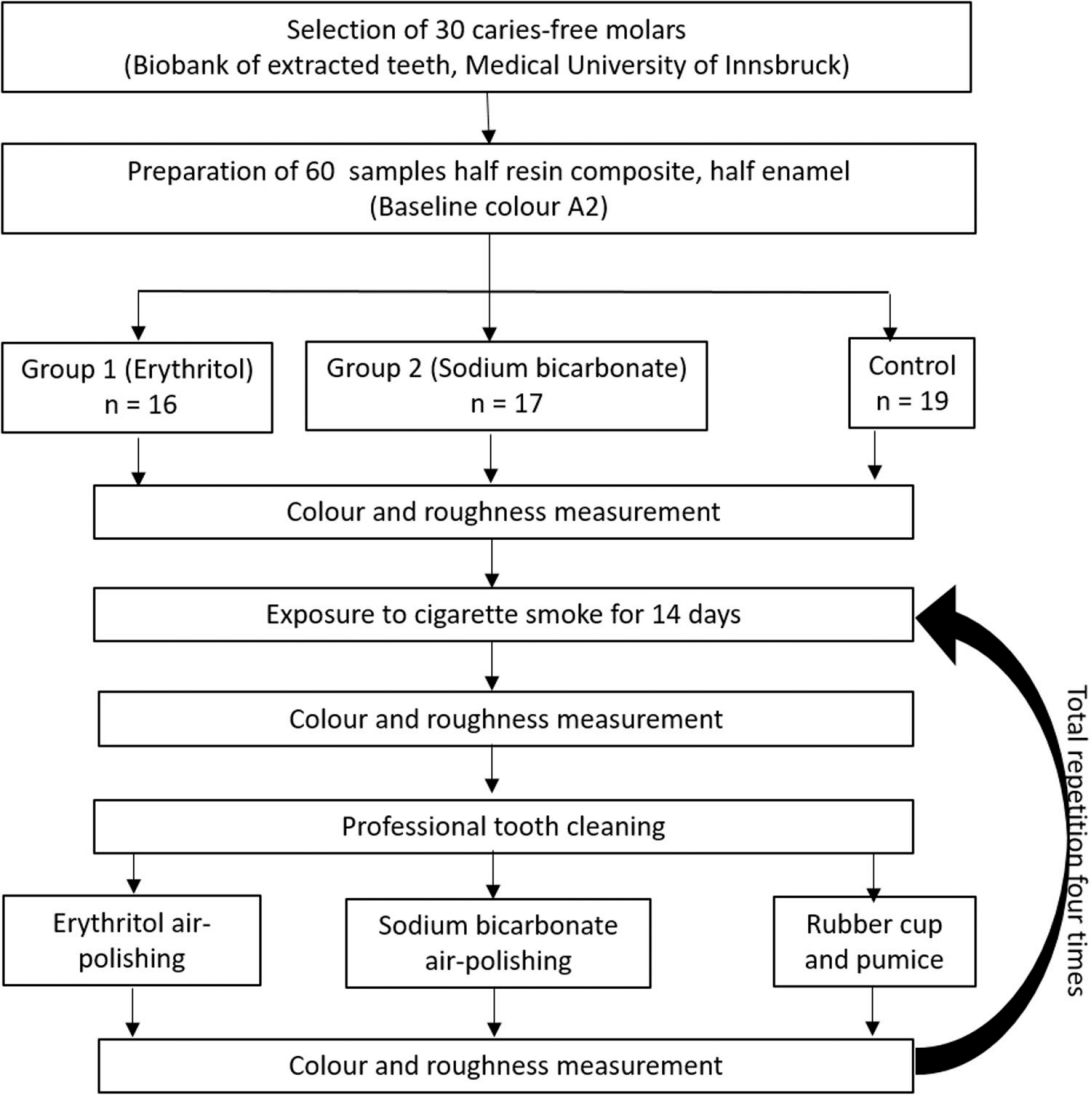
Flowchart of the experimental procedure. After preparation and storing the samples in sterile saliva for 48 h, they were assigned to three groups: Cleaning with minimal abrasive powder devices (MAPD) and erythritol, MAPD and sodium bicarbonate, and cleaning with a rubber cup and pumice stone as a control. Baseline color and surface roughness were recorded before exposing the samples to cigarette smoke (five cigarettes per day) in the smoking chamber for 14 days. After 14 days the samples were cleaned according to their assigned treatment, color and surface roughness were measured before and after the cleaning procedure. The 14‐day smoke exposure and subsequent measurements and cleaning were repeated three times for all samples.

Color and surface roughness measurements were carried out both pre‐ and post‐treatment, and the whole experiment was independently repeated three times (see Figure 2).

### 2.5 Statistical Analysis

The statistical analysis was performed by using IBM SPSS Statistics V.29.0.0.0 (IBM Armonk; NY, USA). The sample size calculation based on the results of our previous study (mean ∆*E* [SD] 8.51 [4.41] for erythritol, 8.04 [3.68] for sodium bicarbonate and 13.08 [5.05] for pumice stone) revealed a number of 14 specimens per group (Sigwart et al. 2024). With 20% drop out, we have rounded up to a samples size of 20 samples per group. Enamel color data followed a normal distribution, while resin composite color and surface roughness data deviated from normality, according to the Kolmogorov–Smirnov test. However, all groups exhibited equal variances. Median and interquartile range were used for descriptive analysis, if not stated otherwise. Significance tests were calculated by Kruskal–Wallis test for a comparison between the three groups. For the direct comparison between two groups, the Mann–Whitney *U* test was performed, and for the different measurement times, a paired, non‐parametric test (Wilcoxon‐signed‐ranked‐test) was used for significance tests. Significance level was set at *α* = 0.05 and the power was set at 0.80.

## 3 Results

### 3.1 Changes of Resin Composite Color

Baseline spectrophotometric analysis revealed a statistically significantly darker median color for the resin composite specimens of test group II (sodium bicarbonate) with median *E* [IQR] = 82.88 [81.02–84.50], compared to *E* 87.87 [84.15–89.64] for test group I (erythritol) and *E* = 86.26 [83.19–88.22] for control (*p* = 0.011), all of which corresponding to a baseline color A2 of the VITA classical shade guide. After the fourth cycle, eight specimens—four samples of the erythritol group, three samples of the sodium bicarbonate group, and one of the control group—were lost due to fracture, independent of the cleaning methods, of the resin composite filling from the enamel specimen.

**Figure 3:**
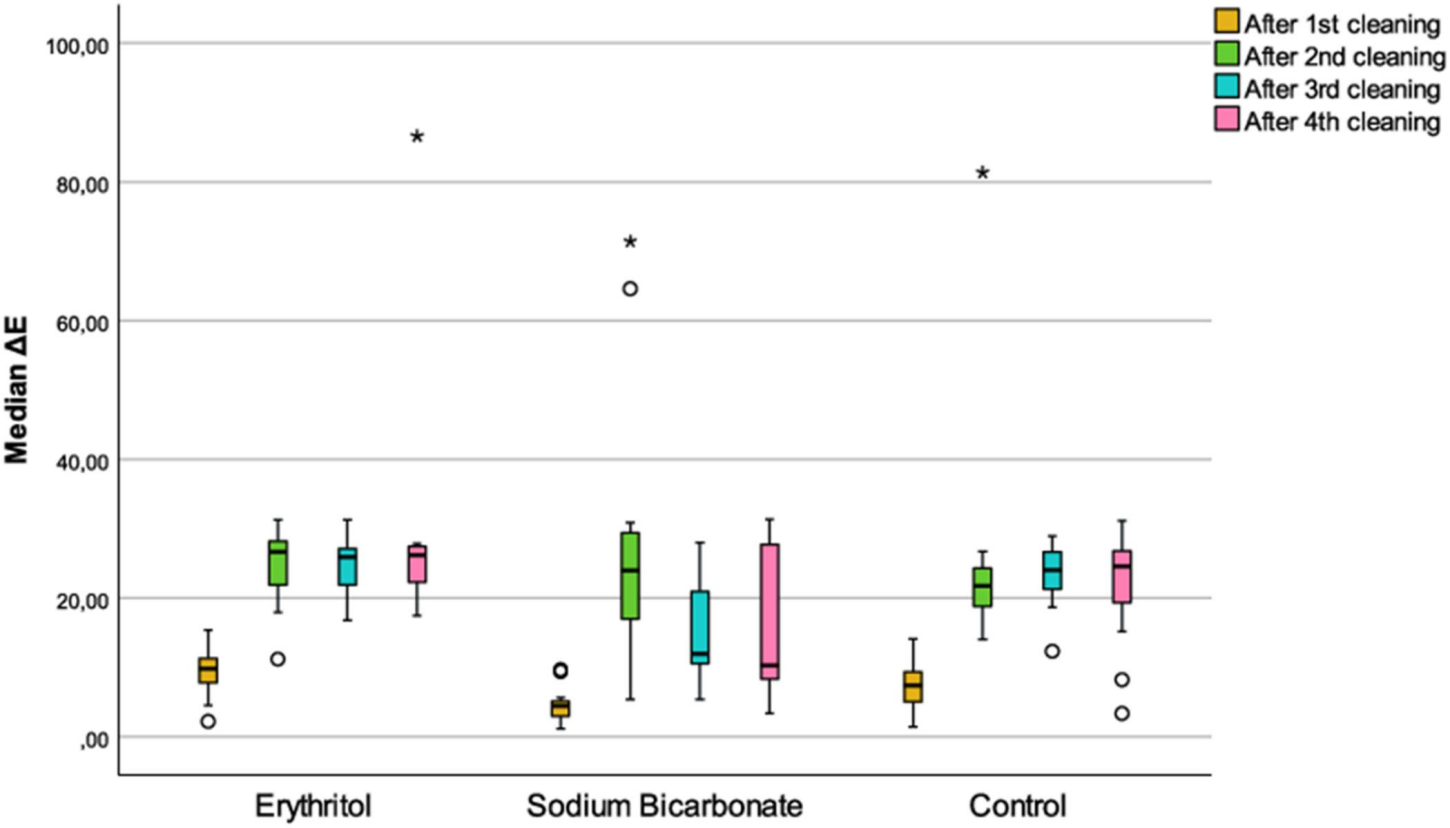
Color measurements of the resin composite of each test and control group. The median values of ∆*E* (y‐axis) are shown in the boxplots with the interquartile range (IQR).

During the first cycle of 14 days of in vitro smoking, the resin composite samples of all three groups darkened statistically significantly compared to baseline with a mean Δ*E* [SD] of 26.34 [1.09] (*p* < 0.001). After subsequent cleaning, the color of resin composite remained statistically significantly darker in all groups compared to baseline (*p* < 0.001) with the greatest median Δ*E* [IQR] 8.82 [5.68–11.08] after air‐polishing with erythritol, the least color difference of Δ*E* = 4.30 [2.82–5.53] with sodium bicarbonate, and 6.95 [4.57–9.39] with rubber cup and pumice (Table 1). The pairwise comparison of the cleaning efficacy revealed no statistically significant difference between the control group and the erythritol group (*p* = 0.296), but between the erythritol and sodium bicarbonate group (*p* < 0.001) and the control group versus sodium bicarbonate (*p* = 0.002) after the first cycle. Nevertheless, the original resin composite color was not achieved in any group with a statistically significant difference of the color *E* compared to baseline (*p* < 0.001) (Table 1; Figure 3).

**Table 1:** Color changes of resin composite samples after each cycle of smoking and polishing compared to baseline. None of the polishing procedures could restore the baseline color at any time. Considering the cleaning performance, although the sodium bicarbonate group revealed a statistically significant difference compared to the other groups, there were no statistically significant differences between the test and the control groups after four cycles. However all groups revealed a statistically significant difference between baseline and final color *E* (*p* = 0.012).

Resin composite	Color difference Δ*E* to baseline; median [IQR]			
	1st Cycle	2nd Cycle	3rd Cycle	4th Cycle
Air‐polishing erythritol (*n* = 20)	8.82 [5.68–11.08]	26.09 [20.78–27.72]	25.61 [21.58–26.97]	26.17 [21.62–27.52]
Air‐polishing sodium‐bicarbonate (*n* = 20)	4.30* [2.82–5.53]	24.28 [17.99–30.49]	13.09 [9.22–22.90]	10.28 [7.44–27.77]
Rubber cup and pumice (*n* = 20)	6.95 [4.57–9.39]	21.11 [18.26–24.52]	23.53 [20.94–26.64]	24.56 [19.22–26.77]

* *p* < 0.05.

Especially after the second cycle, all three groups showed a similar cleaning performance, which continued after the third cycle in the erythritol and control group, but not the sodium bicarbonate group (Table 1). Looking in more detail at the color changes in the sodium bicarbonate group, the median ∆*E* of the resin composite increased after the first cycle to a similar extent as in the erythritol and pumice groups but markedly decreased after the second cycle and remained at this lower level after the third and fourth cycle with a great deviation of the IQR of ∆*E* (IQR 7.44–27.77) (Table 1). After the four cycles of cleaning, all groups showed in general a similar cleaning performance again with no statistically significant difference (*p* = 0.194). Therefore, the difference in color change was Δ*E* [IQR] 26.17 [21.62–27.52] for erythritol, 10.28 [7.44–27.77] for sodium bicarbonate and 24.56 [19.22–26.77] for rubber cup and pumice, with no statistically significant difference between all groups in final colors *E* (*p* = 0.114), but a statistically significant difference compared to baseline (*p* = 0.012).

When the color changes Δ*E* of resin composite specimens were translated to the VITA classical shade guide, the Δ*E* values of the erythritol and control groups corresponded to a color change from VITA A2 to C4. In contrast, median ΔE of the sodium bicarbonate group exhibited a smaller color change corresponding to a shift from VITA A2 to A3.5. The color difference between the erythritol and the control groups was below the AT (∆*E* = 1.61), indicating that over 50% of the patients would not perceive a difference whether the specimen was cleaned with erythritol or with pumice paste. In contrast, the median ∆*E* of the sodium bicarbonate group deviated strongly from that of the erythritol group, exceeding the AT.

During the first two smoking cycles, the ∆*E* values of the erythritol and control groups exhibited a linear deviation, followed by a plateau in the resin composite samples. In contrast, the sodium bicarbonate group demonstrated a smaller deviation from the baseline color after four cycles, although with a greater interquartile range (IQR) compared to baseline.

### 3.2 Changes of Enamel Color

The detailed results of the color differences ∆*E* of enamel are presented in the supplements (see Table S1). In brief, for the cleaning efficacy of enamel, there was no statistically significant difference in Δ*E* between erythritol, sodium bicarbonate air‐polishing and the control group after four cycles of smoking and cleaning compared to baseline (Δ*E* = 15.32 [9.68–24.08], 17.44 [7.14–22.55] and 17.58 [15.90–19.71] respectively; *p* = 0.515), but none of the polishing procedures could restore the original color.

Furthermore, the median Δ*E* of enamel in the erythritol and control group after four cycles was statistically significantly lower than that of resin composite specimens (*p* < 0.001 for erythritol vs. *p* = 0.015 for control), indicating a more pronounced darkening of composite restorations compared to enamel.

### 3.3 Comparison of Color Changes Between Enamel and Resin Composite Restorations

In a second step, the color differences ∆*E*_ER_ between enamel and each corresponding resin composite sample were evaluated after exposure to cigarette smoke and after each cleaning procedure (see Table 2).

**Table 2:** Color difference between enamel and resin composite samples after each cleaning procedure. The average color difference median ∆*E*_ER_ [IQR] between the enamel and resin composite samples was calculated at baseline and after each smoking cycle. There were no statistically significant differences between the three groups after each cleaning cycle (*p* > 0.05).

	Color difference Δ*E*_ER_ between enamel and resin; median [IQR]				
	Baseline	1st Cycle	2nd Cycle	3rd Cycle	4th Cycle
Air‐polishing erythritol	9.24 [6.07–13.39]	12.71 [6.55–15.45]	10.23 [6.28–17.65]	9.02 [5.83–13.70]	14.16 [6.01–17.84]
Air‐polishing sodium‐bicarbonate	11.59 [7.45–15.29]	9.78 [6.88–17.46]	11.40 [6.83–24.06]	9.70 [5.34–13.17]	12.17 [10.13–17.36]
Rubber cup and pumice	11.11 [6.83–15.03]	12.36 [9.07–16.19]	7.63 [5.83–13.38]	7.04 [4.49–13.91]	12.46 [8.87–17.28]

After four smoking and cleaning cycles the color difference ∆*E*_ER_ between enamel and resin composite increased in all groups (see Table 2). While the erythritol group showed the greatest increase of ∆*E*_ER_ compared to baseline, ∆*E*_ER_ in the sodium bicarbonate and control group increased also, however with no statistically significance in all groups (*p* = 0.721 vs. *p* = 0.609 vs. *p* = 0.295). From a clinical perspective, the erythritol group showed the greatest color difference between baseline and after four cleaning cycles being above the AT, while the color changes of the sodium bicarbonate and the control group were under the PT.

### 3.4 Surface Roughness of Enamel and Resin Composite

There were no statistically significant differences in *R*_z_ between the groups baseline, neither for enamel nor for resin composite specimens (*p* = 0.319 for enamel vs. *p* = 0.127 for resin composite). On the resin composite surfaces all three cleaning procedures led to a statistically significant increase in roughness (*R*_z_) after four cycles (Table 3). Erythritol exhibited the least increase with no statistically significant difference from baseline median *R*_z_ [IQR] = 1.60 [1.18–2.18] to 2.26 [1.80–3.60] after four cycles (*p* = 0.133). While the control group increased statistically significant from baseline *R*_z_ = 0.99 [0.51–1.89] to 1.91 [1.52–2.57] after four cycles (*p* = 0.010), the sodium bicarbonate group revealed the greatest increase with a statistically significant difference of the roughness *R*_z_ from baseline *R*_z_ = 1.21 [0.65–1.94] to 4.45 [3.97–5.52] after four smoking and cleaning cycles (*p* = 0.003).

**Table 3:** Roughness measurements before and after four cycles of smoking and cleaning. Surface roughness of enamel was not statistically significantly different after four cycles of smoking and cleaning, neither for air‐polishing with erythritol or sodium‐bicarbonate nor for the control group. On the resin composite samples, statistically significantly higher *R*_z_‐values were measured after four cycles in the sodium bicarbonate and control group, but not in the erythritol group. The sodium bicarbonate group showed a high increase in *R*_t_ of the luting gap, compared to the erythritol or control group, which differed highly statistically significant in comparison to the other groups (*p* < 0.001).

*R*_z_	Enamel		
	Baseline	Final	*p*‐value
Erythritol (*n* = 20)	2.74 [1.68–3.70]	2.66 [2.07–4.08]	0.551
Sodium Bicarbonate (*n* = 20)	2.07 [1.32–3.12]	3.63 [1.72–4.40]	0.283
Control (*n* = 20)	1.90 [1.20–2.79]	2.32 [1.47–2.90]	0.747

***R*_z_**	**Composite**		
	**Baseline**	**Final**	***p*‐value**
Erythritol (*n* = 20)	1.60 [1.18–2.18]	2.26 [1.80–3.60]	0.005
Sodium Bicarbonate (*n* = 20)	1.21 [0.65–1.94]	4.45 [3.97–5.52]	< 0.001
Control (*n* = 20)	0.99 [0.51–1.89]	1.91 [1.52–2.57]	0.043

***R*_t_**	**Luting gap**		
	**Baseline**	**Final**	***p*‐value**
Erythritol (*n* = 20)	15.37 [9.92–32.87]	17.28 [12.58–36.71]	0.375
Sodium Bicarbonate (*n* = 20)	12.71 [5.66–31.67]	22.31 [19.07–33.39]	< 0.001
Control (*n* = 20)	10.03 [5.51–18.03]	7.18 [2.47–26.52]	0.007

In contrast, enamel surface roughness *R*_z_ did not differ significantly from baseline after four cycles of smoking and air‐polishing, regardless of whether erythritol or sodium bicarbonate powders were used, or in the control group (Table 3). Specifically, for erythritol, median *R*_z_ [IQR] values after four cycles exhibited a slight decrease from 2.74 [1.68–3.70] to 2.66 [2.07–4.08] (*p* = 0.814) compared to the baseline. Conversely, a slight increase in roughness (*R*_z_) was observed after four cycles in both, the sodium bicarbonate group (from 2.07 [1.32–3.12] to 3.63 [1.72–4.40]) and the control group (from 1.90 [1.20–2.79] to 2.32 [1.47–2.90]), though these changes were not statistically significant (*p* = 0.131 vs. *p* = 0.492).

**Figure 4:**
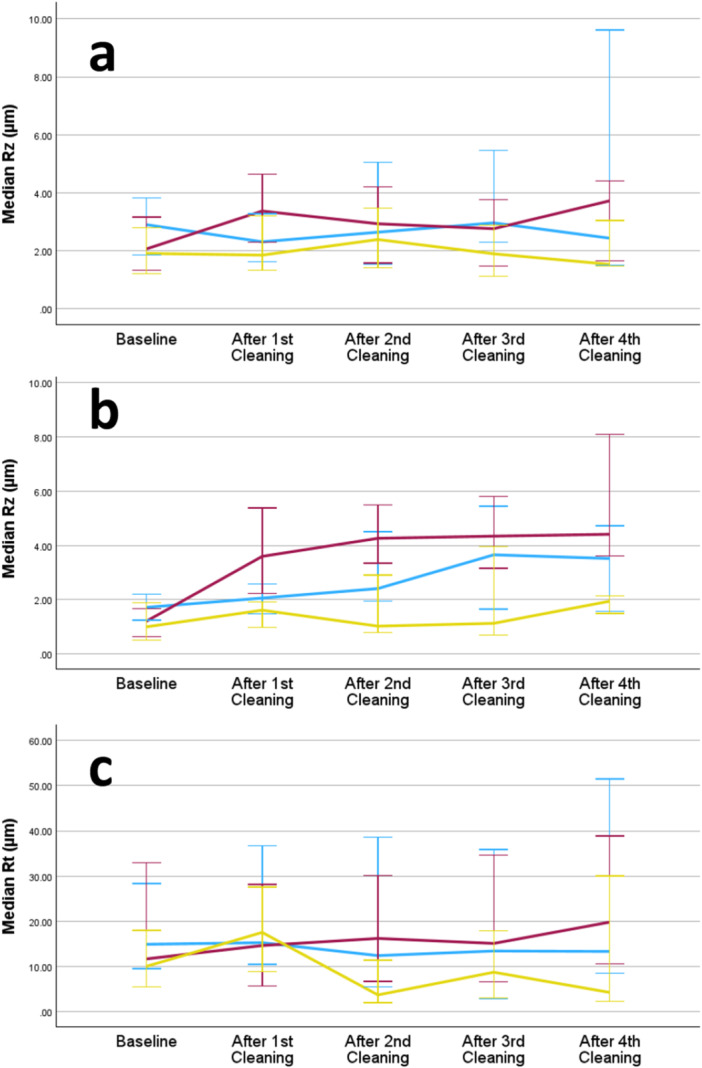
Roughness measurement of enamel (a), resin composite (b), and luting gap (c). The median *R*_z_ values for enamel and resin composite, along with the median *R*_t_ values for the luting gap and their corresponding confidence intervals (CI; 95%), are presented in the figures. The blue line represents the erythritol group, the red lines the sodium bicarbonate and the yellow lines the control group. On enamel surfaces, *R*_z_ did not increase significantly in any of the three groups.

On the luting gap, the erythritol group led to a slightly but not statistically significant increase of *R*_t_ from baseline median [IQR] *R*_t_ = 15.37 [9.92–32.87] to 17.28 [12.58–36.71] after four cycles (*p* = 0.695) (Table 3; Figure 4). Conversely, the control group showed a non‐statistically significant decrease of *R*_t_ from baseline 10.03 [5.51–18.03] to 7.18 [2.47–26.52] (*p* = 0.758). The most substantial difference was detected in the sodium bicarbonate group where *R*_t_ increased from Baseline *R*_t_ = 12.71 [5.66–31.67] to 22.31 [19.07–33.39] (*p* = 347) after four cycles (Figure 4). In comparison to the erythritol and control groups the great increase of the sodium bicarbonate group is more evident as it shows a highly statistically significant increase compared to the other groups (*p* < 0.001), where no statistically significant difference detected (*p* = 0.953 vs. *p* = 0.620).

## 4 Discussion

The removal of tobacco stains is both time‐consuming and challenging, especially when dealing with surface irregularities, fissures and small indentations on teeth. In the authors' experience, the removal of tobacco stains is a major motivation for many smoking patients to seek regular professional dental cleaning, regardless of the dental health factor. However, it is crucial to consider the potential abrasiveness of powder devices with the most appropriate cleaning efficacy, when repeatedly used on enamel or resin composite surfaces during maintenance therapy (Wolgin et al. 2021; Eickholz et al. 2008; Pretzl et al. 2008; Axelsson et al. 2004). In this study, we aimed to investigate the color changes of resin composite restorations caused by moderate smoking and evaluate the efficacy of different cleaning methods and their impact on surface roughness. We designed an in vitro smoking chamber to create standardized tobacco stains and evaluate the cleaning efficacy, in terms of color and roughness change, between erythritol and sodium bicarbonate air‐polishing and cleaning with rubber cup and pumice.

The null hypothesis was confirmed, as no statistically significant differences in ∆*E* were observed in the color of resin composite restorations between conventional polishing with a rubber cup and pumice stone and air‐polishing with erythritol or sodium bicarbonate, even after repeated use. Therefore, it can be stated that erythritol is as effective as the control procedures in removing tobacco stains from resin composites, although none of the procedures could restore the baseline color with an increase of median ∆*E* [IQR] = 26.17 [21.62–27.52] for erythritol and ∆*E* = 24.56 [19.22–26.77] for pumice stone. It should be noted that the median ∆*E* value of the sodium bicarbonate group increased less markedly after four cycles of smoking and air‐polishing (median ∆*E* [IQR] = 10.28 [7.44–27.77]), indicating a closer return to the baseline color after polishing compared to the other groups, probably due to the high abrasiveness of the powder. However, due to the substantial interquartile range of color values, the difference was not statistically significant (see Table 1; Figure 3). Also on enamel, air‐polishing with erythritol proved to be as effective in removing tobacco stains as air‐polishing with sodium bicarbonate and cleaning with a rubber cup and pumice. These findings are consistent with the results of a previous investigation by the same authors, which examined the cleaning efficacy of enamel and dentine samples (Sigwart et al. 2024).

Clinically relevant is that none of the applied cleaning methods were able to restore the baseline color, neither of enamel nor of resin composite. Thus, in vitro smoking of the samples resulted in a persistent darkening of the specimens, and darkening of resin composite samples was more pronounced than of enamel. This phenomenon is also postulated by the meta‐analysis of Karanjkar et al. where the literature suggested a greater discoloration of dental resin composites compared to dental hard tissues (Karanjkar et al. 2023). Based on the present in vitro study, it must be assumed that the varying degree of color change of enamel and composite is clinically perceptible, since the color difference Δ*E*_ER_ between enamel and resin composite was above the perceptibility and acceptability threshold for color differences, PT Δ*E* = 1.2 and AT Δ*E* = 2.7 (Rioseco and Wagner 2021; Paravina et al. 2015; Khashayar et al. 2014). Therefore, if smoking cessation is no option for patients and color difference is unacceptable, other types of restorations, for example ceramic restorations, should be taken faster into consideration.

The surface roughness of enamel treated with erythritol and those in the control group remained nearly unchanged after four cycles of smoking and cleaning, which is consistent with the data of our previous study (Sigwart et al. 2024). Interestingly, surface roughness of resin composite specimens increased statistically significant in the sodium bicarbonate and control group, but not after air‐polishing with erythritol (*p* = 0.133), indicating that minimally abrasive powders can lead to the least alterations of composite restoration surfaces. Although the erythritol group shows a minimal alteration, the clinical relevance of the observed increase in surface roughness remains debatable. However, further studies should determine whether repolishing resin composite fillings with dedicated instruments after air‐polishing with erythritol is necessary. The sodium bicarbonate group exhibited a highly statistically significant increase compared to erythritol (*p* < 0.001) from *R*_z_ 1.21 [0.65–1.94] at baseline to *R*_z_ 4.45 [3.97–5.52] post fourth cycle, which is why the use of this powder on resin composite restorations must be considered contraindicated.

Another question we addressed was how air‐polishing affects the luting gap. In the air polishing group using erythritol, there was no statistically significant increase of the luting gap *R*_t_ after four cycles of smoking and cleaning (*p* = 0.695), whereas in the control group, *R*_t_ decreased from baseline 10.03 [5.51–18.03] to 7.18 [2.47–26.52], likely due to the luting gap becoming filled with pumice debris or due to the abrasiveness of the method. Air‐polishing with sodium bicarbonate, however, resulted not in a statistically significant increase of the luting gap (baseline *R*_t_ = 12.71 [5.66 −31.67] vs. post fourth cycle *R*_t_ = 22.31 [19.07–33.39]; *p* = 0.347) which could probably traced back to the limitation of a non‐parametric test that has to be used due the absence of a normal distribution. However, when compared to the erythritol and group the difference is more visible, as there was a highly significant difference (*p* < 0.001). In the author's opinions, sodium bicarbonate must therefore be considered as clearly contraindicated for use on resin composite restorations.

Mukatash Nimri (2015) highlighted the variability in the abrasiveness of pumice stone, which can be attributed to factors such as particle size, concentration, and applied surface pressure. This observation prompts a critical question regarding the potential wear of enamel and resin composite materials resulting from professional tooth cleaning procedures involving pumice. In the present study, pumice stone was employed as a standard control method for the effective removal of dental discolorations. Given the unpredictability of wear associated with pumice use, minimally abrasive prophylaxis devices (MAPD) should be generally preferred, as sodium bicarbonate or hand instruments have also demonstrated high abrasiveness in previous research (Petersilka, Bell et al. 2003; Atkinson et al. 1984; Sigwart et al. 2024; Kruse et al. 2024). A number of studies examined the effectiveness of air polishing on resin composites, concluding glycine powders showing promising results in reduced wear compared to sodium bicarbonate or conventional polishing (Güler et al. 2010, 2011; Janiszewska‐Olszowska et al. 2020; Babina et al. 2021; Salerno et al. 2010; Shimizu et al. 2014; Tamura et al. 2017).

A notable strength of our study is the reproducibility of the standardized protocol for the assessment of tooth discoloration, as evidenced by the consistent findings on enamel color across the present study and previous investigations (Sigwart et al. 2024). Paolone et al. (2022) described the importance of standardization, with a comparable protocol to our own in this field of research, in order to achieve replicable results. Numerous previous studies have exposed specimens to 20 cigarettes; however, the smoking chamber used in this study ensures a uniform smoke dispersion, creating discolorations that are analogous to those observed in vivo already with five cigarettes. A limitation might be that the resin composite samples did not exhibit a normal distribution at baseline, despite using the same resin composite of shade A2. In general, the color determination using spectral photometry is a more reproducible method for measuring color than either a visual examination by a dental professional or the patient's own view (Derdilopoulou et al. 2007). Tabatabaian et al. (2021) described the high accuracy in shade determination of the spectrophotometer. We preferred this device to calculate ∆*E* from the L*a*b‐color space as well as the clinical shade for a comparison to a clinical setting. Since all samples were measured with the same device, the color differences within the study should be reliable. Furthermore, it should be stated that measuring the color with a spectroradiometer could reveal more perceptible differences, apart from the lack of clinical significance, which is described by Akl et al. (2022).

As a notable limitation, the extent of wear of dental hard tissues or resin composite restorations was not assessed in the present study. In fact, profilometric measurements revealed a statistically significant increase of the roughness of the resin composite samples and the luting gap, but further investigations such as optical coherence tomography or quantitative light‐induced fluorescence could reveal the wear and a better understanding of the dimension of the defects on the samples caused by air polishing. Within their limitations and the lack of investigations on restoration materials, they show promising results on dental hard tissues and should not be disregarded (Huysmans et al. 2011; Lussi and Carvalho 2014).

## 5 Conclusion

Based on our data, air‐polishing with erythritol can be recommended as a safe and effective method for removing tobacco stains from resin composite restorations. Although all groups showed a detectable increase in surface roughness, sodium bicarbonate resulted in the highest increase of both, surface roughness of the resin composite and the luting gap. It should therefore be considered as contraindicated on resin composite restorations. None of the investigated procedures were able to restore the original color and all led to an increased color difference between enamel and resin composite, which could unavoidably lead to a non‐acceptable or unpredictable esthetic result.

## Author Contributions

Lukas Sigwart and Ines Kapferer‐Seebacher contributed to the study conception and design and funding acquisition. Material preparation and data collection were supervised by Lukas Sigwart. The analysis of the data was conducted by Vera Wiesmüller. The first draft of the manuscript was written by Lukas Sigwart. All authors revised and approved the final manuscript.

## Ethics Statement

The Ethics committee of the Medical University of Innsbruck, Austria, approved the study (EK 1027/2023). The study was conducted per the Helsinki Declaration of 1964 and its later amendments.

## Consent

Patients who donated their extracted teeth for scientific research signed a consent form before tooth preservation.

## Conflicts of Interest

The authors declare no conflicts of interests.

## Supporting information

Table S1: Colour changes of enamel samples after each cycle of smoking and polishing compared to baseline.

## Data Availability

The data that support the findings of this study are available from the corresponding author upon reasonable request.
