# Supplementary material for: Cleaning Efficacy of Air Polishing on Tobacco‐Stained Resin Composite: An In‐Vitro Study
Source: Clin Exp Dent Res. 2026 Jul 8;12(4):e70406. doi: 10.1002/cre2.70406 (PMC13345598; doi:10.1002/cre2.70406)
Supplement: Supplementary file 1 — Table S1: Colour changes of enamel samples after each cycle of smoking and polishing compared to baseline. [file CRE2-12-e70406-s001.docx]

***Supplements***

**Table S1. Colour changes of enamel samples after each cycle of smoking and polishing compared to baseline.** The colour difference ΔE is given as median [IQR] and was calculated between baseline and the end of each cycle. The higher ΔE, the greater is the colour difference after each cycle to baseline. None of the polishing procedures could restore the enamel baseline colour at any time.

| **Enamel** | **Colour difference ΔE to baseline; median [IQR]** | | | |
| --- | --- | --- | --- | --- |
|  | **1st Cycle** | **2nd Cycle** | **3rd Cycle** | **4th Cycle** |
| **Air-polishing erythritol (n = 20)** | 10.73 [6.17 - 14.46] | 19.61 [11.35 - 21.92] | 19.62 [10.47 -28.31] | 15.32 [9.68 - 24.08] |
| **Air-polishing sodium-bicarbonate (n = 20)** | 7.14 [4.50 - 12.79] | 12.88 [9.91 - 21.25] | 14.20 [7.78 - 25.59] | 17.44 [7.14 - 22.55] |
| **Rubber cup and pumice (n = 20)** | 6.56 [3.63 - 10.56] | 14.65 [10.69 - 22.69] | 15.11 [11.42 - 22.38] | 17.58 [15.90 - 19.71] |
